# Supplementary material for: Injury-induced Erk1/2 signaling tissue-specifically interacts with Ca2+ activity and is necessary for regeneration of spinal cord and skeletal muscle
Source: Cell Calcium. Author manuscript; Available in PMC 2022 Oct 7. (PMC9542431; doi:10.1016/j.ceca.2022.102540)
Supplement: Suppl Figures — Figure S1. Mek1/2 or Shp2 inhibition reduces active Erk1/2 in injured and regenerating tail. Stage-39 larvae were incubated immediately following amputation with either Mek1/2 (A; 3 μM PD0325901) or Shp2 (B; 50 μM SHP099) inhibitor or only vehicle (0.1 or 0.5% DMSO; control) for 24–26 h at 21 °C, and then processed for whole-mount, pErk1/2 immunostaining. Images are representative maximum-intensity projections. Scale bars: 100 μm. a: anterior, p: posterior. Figure S2. Assessment of tissue regeneration in amputated larvae. Stage-39 larvae were amputated, allowed to regenerate for 1 to 5 days, then fixed and processed for whole-mount immunostaining for tissue-specific markers. Images are representative maximum-intensity projections. Arrowheads indicate the site of amputation. A, Colocalization of pan-neuronal cell membrane marker (Xen1) and neural stem cells (Sox2) at 3 dpa. B, Larvae were incubated following amputation with either vehicle-only (0.1% DMSO; control) or Mek1/2 inhibitor (10 μM PD98059) and organized muscle chevrons were counted under brightfield illumination 30 min post-amputation and again before fixation at 3 days post-amputation (dpa). Data are mean±SEM number of organized chevrons; N = 5 experiments with n = 7–9 larvae per treatment per experiment. C, Muscle chevron organization through 5 dpa shown by 12/101 staining (mature skeletal muscle). The purple line delineates a single, organized muscle chevron. The region measured for regenerated skeletal muscle is outlined in red. D, The amputation site (white arrowheads) was determined by notochord staining (Tor70) and morphology, and the length of spinal cord (red, dashed line; Sox2) and notochord were measured from this site. E, Quantification of the regeneration timecourse for spinal cord (solid line; Sox2) and muscle (dashed line; 12/101) regeneration during the first 3 days at 21 °C after amputation at stage 39. N ≥ 3 experiments with n = 3–6 larvae per treatment per experiment. Scale bars: 50 (A) o [file NIHMS1837153-supplement-Suppl_Figures.pdf]

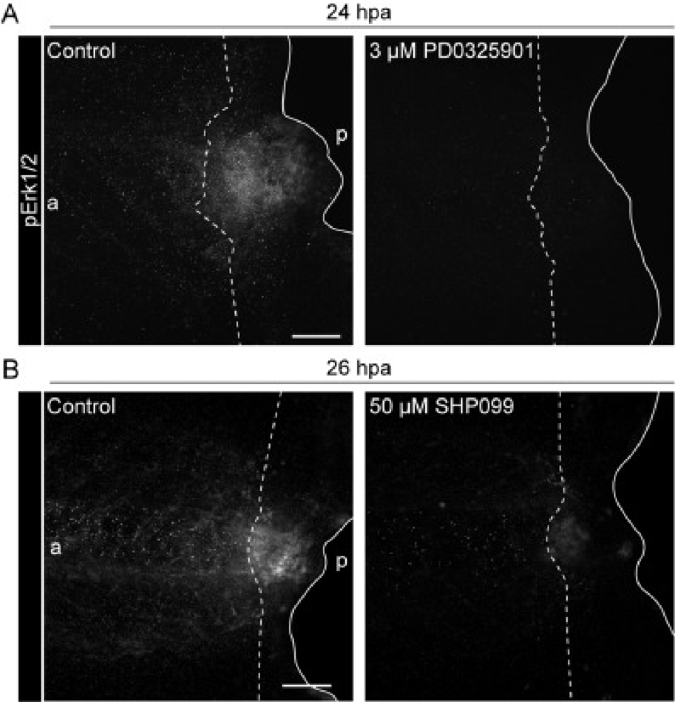
A
24 hpa
Control
3 µM PD0325901
pErk1/2
a
p
B
26 hpa
Control
50 µM SHP099
a
p

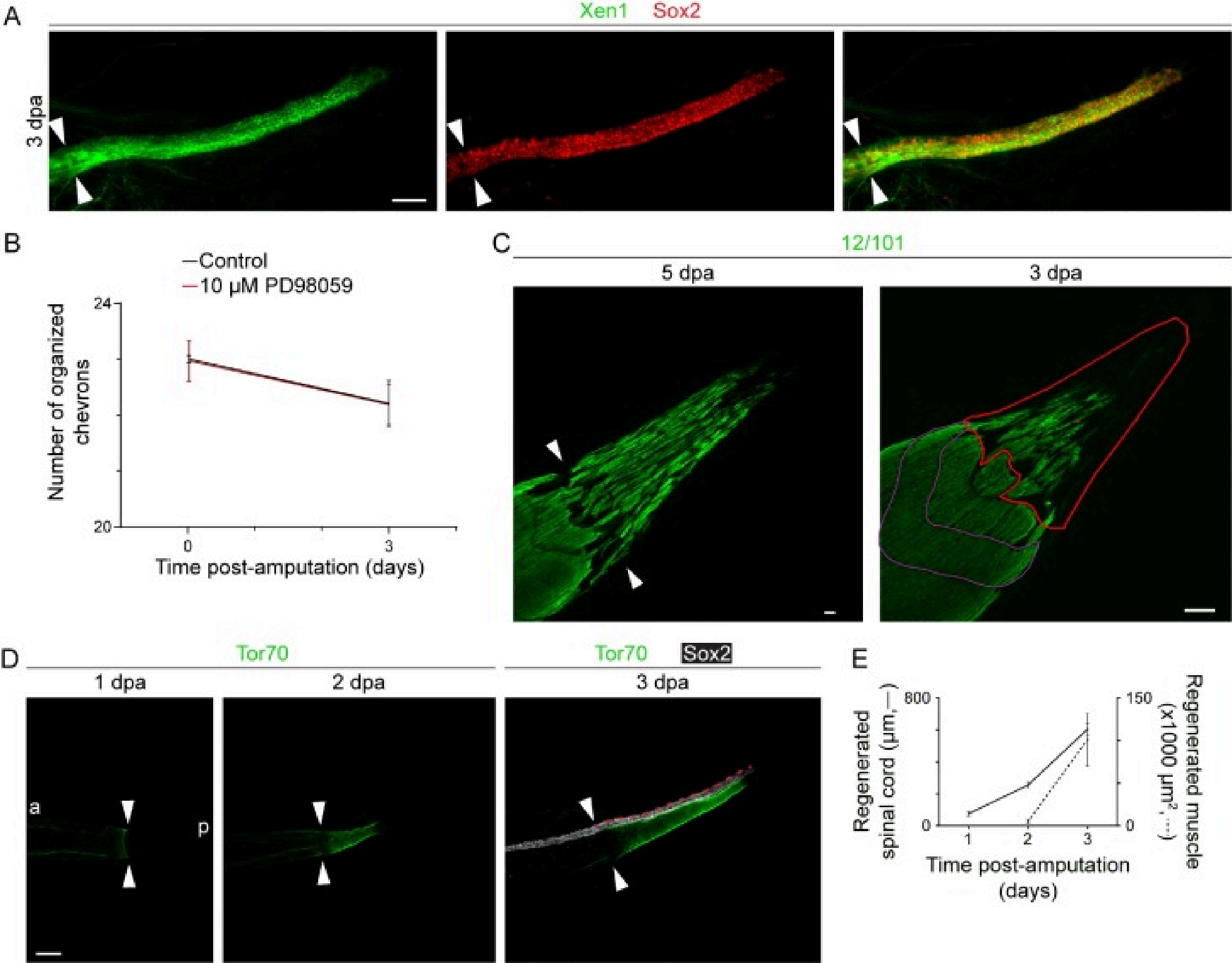

A
Xen1 Sox2
3 dpa
B
Control
10 μM PD98059
Number of organized chevrons
24
20
0
3
Time post-amputation (days)
C
12/101
5 dpa
3 dpa
D
Tor70
1 dpa
2 dpa
Tor70 Sox2
3 dpa
a
p
E
Regenerated spinal cord (μm, —)
Regenerated muscle (x1000 μm², ····)
800
0
150
0
1
2
3
Time post-amputation (days)

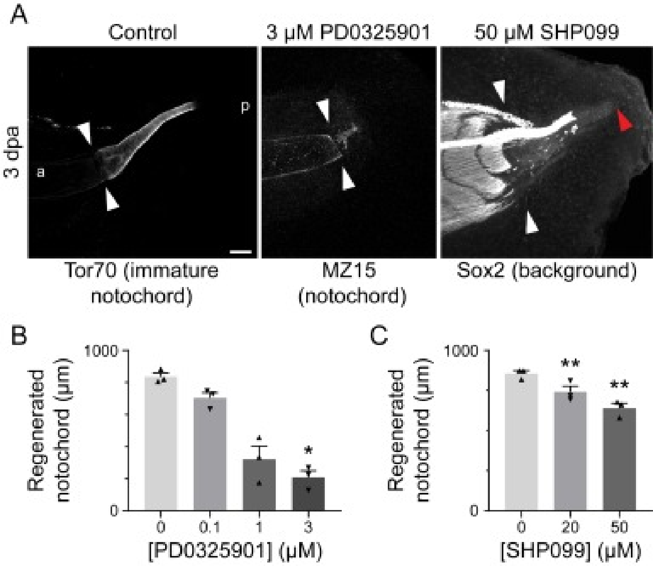

A
Control
3 µM PD0325901
50 µM SHP099
3 dpa
p
a
Tor70 (immature notochord)
MZ15 (notochord)
Sox2 (background)
B
Regenerated notochord (µm)
1000
0
0
0.1
1
3
*
[PD0325901] (µM)
C
Regenerated notochord (µm)
1000
0
0
20
50
**
**
[SHP099] (µM)

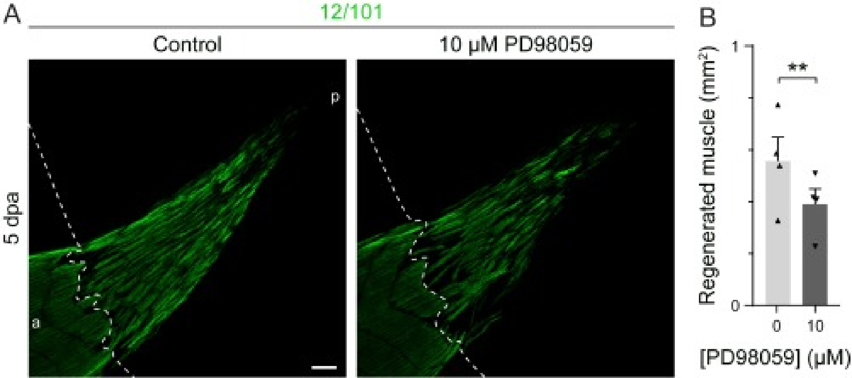
A
12/101
Control
10 µM PD98059
5 dpa
p
a
B
Regenerated muscle (mm²)
**
1
0
0
10
[PD98059] (µM)

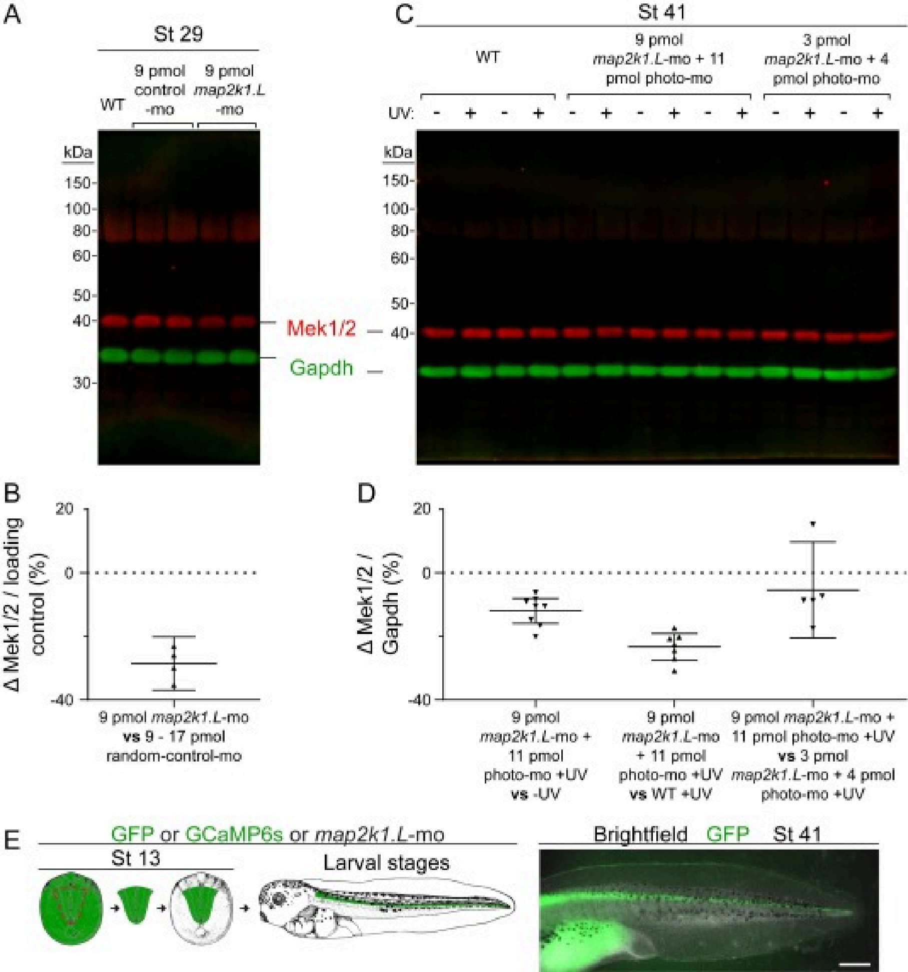

A
St 29
WT
9 pmol control -mo
9 pmol map2k1.L -mo
kDa
150
100
80
60
50
40
30
Mek1/2
Gapdh
C
St 41
WT
9 pmol map2k1.L-mo + 11 pmol photo-mo
3 pmol map2k1.L-mo + 4 pmol photo-mo
UV: - + - + - + - + - + - + - + - +
kDa
150
100
80
60
50
40
B
Δ Mek1/2 / loading control (%)
20
0
-40
9 pmol map2k1.L-mo vs 9 - 17 pmol random-control-mo
D
Δ Mek1/2 / Gapdh (%)
20
0
-40
9 pmol map2k1.L-mo + 11 pmol photo-mo +UV vs -UV
9 pmol map2k1.L-mo + 11 pmol photo-mo +UV vs WT +UV
9 pmol map2k1.L-mo + 11 pmol photo-mo +UV vs 3 pmol map2k1.L-mo + 4 pmol photo-mo +UV
E
GFP or GCaMP6s or map2k1.L-mo
St 13
Larval stages
Brightfield GFP St 41

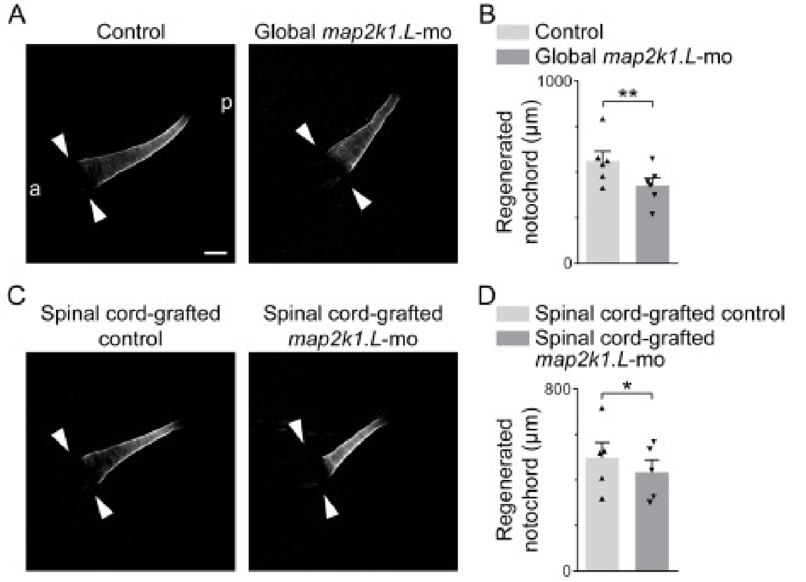
A
Control
Global map2k1.L-mo
p
a
B
Control
Global map2k1.L-mo
1000
**
0
Regenerated notochord (µm)
C
Spinal cord-grafted control
Spinal cord-grafted map2k1.L-mo
D
Spinal cord-grafted control
Spinal cord-grafted map2k1.L-mo
800
*
0
Regenerated notochord (µm)

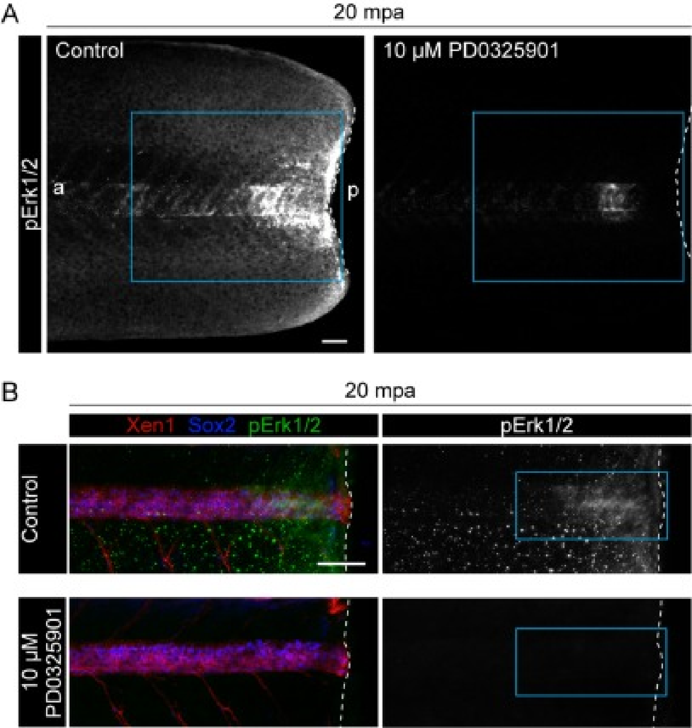
A
20 mpa
pErk1/2
Control
10 μM PD0325901
a
p
B
20 mpa
Xen1 Sox2 pErk1/2
pErk1/2
Control
10 μM PD0325901
